# Tularemia in Alaska, 1938 - 2010

**DOI:** 10.1186/1751-0147-53-61

**Published:** 2011-11-18

**Authors:** Cristina M Hansen, Amy J Vogler, Paul Keim, David M Wagner, Karsten Hueffer

**Affiliations:** 1 Institute of Arctic Biology and Department of Biology and Wildlife, University of Alaska Fairbanks, 902 N. Koyukuk Dr., Fairbanks, AK 99775, USA; 2 Center for Microbial Genetics and Genomics, Northern Arizona University, PO Box 4073, Flagstaff, AZ 86011, USA

## Abstract

Tularemia is a serious, potentially life threatening zoonotic disease. The causative agent, *Francisella tularensis*, is ubiquitous in the Northern hemisphere, including Alaska, where it was first isolated from a rabbit tick (*Haemophysalis leporis-palustris*) in 1938. Since then, *F. tularensis *has been isolated from wildlife and humans throughout the state. Serologic surveys have found measurable antibodies with prevalence ranging from < 1% to 50% and 4% to 18% for selected populations of wildlife species and humans, respectively. We reviewed and summarized known literature on tularemia surveillance in Alaska and summarized the epidemiological information on human cases reported to public health officials. Additionally, available *F. tularensis *isolates from Alaska were analyzed using canonical SNPs and a multi-locus variable-number tandem repeats (VNTR) analysis (MLVA) system. The results show that both *F. t. tularensis *and *F. t. holarctica *are present in Alaska and that subtype A.I, the most virulent type, is responsible for most recently reported human clinical cases in the state.

## Introduction

Tularemia is a serious and potentially life threatening zoonotic disease caused by the Gram-negative bacterium *Francisella tularensis*. Due to its high virulence and zoonotic potential, *F. tularensis *is listed as a category A select bioterrorism agent. *F. tularensis *has been weaponized in the past by the United States, Japan, the former USSR, and potentially other countries [1]. The organism was first isolated from a ground squirrel in 1911 in Tulare County, CA. It was named *Bacterium tularense*, was later reclassified as *Pasteurella tularense*, and finally, in 1966, was named *Francisella tularensis *after Edward Francis. Descriptions of a plague-like disease now considered to be tularemia predate this first isolation, going as far back as 1818 in Japan [2]. The first laboratory-confirmed human case was reported in 1914 [3]. Since then *F. tularensis *has been isolated from more than 250 host species [4].

*F*. *tularensis *is ubiquitous in the Northern hemisphere and currently there are four recognized subspecies. *F. tularensis *subsp. *tularensis *(type A) is the most virulent of subspecies and is found throughout North America. *F. tularensis *subsp. *holarctica *(type B) is less virulent and is found throughout the Northern hemisphere. The distinction between type A and B tularemia was first made in the middle of the 20th century [5]. Type A is divided into types A.I and A.II, and A.I is still further divided into types A.Ia and A.Ib. In a review of isolates collected in the US over 40 years, the highest human mortality rate was associated with type A.Ib (12/49 or 24%), followed by type B (8/108 or 7%), type A.Ia (2/55 or 4%), and finally, type A.II (0/53 or 0%)[6]. The third subspecies, *F. tularensis *subsp. *mediasiatica *is virulent and has been isolated in central Asia. Finally, many consider *F. tularensis *subsp. *novicida *to be a fourth subspecies of *F. tularensis *based on genetics and biochemical requirements [7], though this classification is still disputed [8,9]. *F. tularensis *subsp *novicida *is generally avirulent in humans and is distributed globally [2,10].

The disease caused by *F. tularensis *depends on the route of entry. Ulceroglandular tularemia, the most common form of disease, results from exposure through the skin (either preexisting wound or arthropod bite). This form results in an ulcer at the site of infection followed by lymphadenopathy. Pneumonic tularemia, the most serious form of disease, results from inhalation of aerosolized bacteria. Other forms of the disease include oculoglandular (exposure via the eye), oropharyngeal (ingestion), and typhoidal tularemia (systemic infection without a primary ulcer).

Here we review the history of tularemia in both wildlife and humans in the state of Alaska. We also report on the genetic characterization of recent Alaskan *F. tularensis *human and animal isolates using canonical SNPs (canSNPs) and multi-locus variable tandem repeat (VNTR) analysis (MLVA).

### Tularemia in wildlife in Alaska

In Alaska, *F. tularensis *was first isolated from a rabbit tick (*Haemophysalis leporis-palustris*) removed from a varying hare (*Lepus americanus*) near Fairbanks in 1938 [11]. The isolated strain was virulent in both guinea pigs and rabbits, resulting in enlarged spleens and areas of focal necrosis in both the spleens and livers. The high virulence in both species suggests that the isolate may have been type A. Later, an additional two virulent and likely type A isolates were obtained when suspensions of ground ticks removed from two healthy hares were inoculated into guinea pigs [12,13]. Isolates collected from subsequent animals indicated the presence of a less virulent type, likely type B. The first of these was an isolate obtained from ticks collected from willow ptarmigan (*Lagopus lagopus*) in the Fairbanks area in 1959 [14]. Voles sampled during the summer of 1963 on the Alaska Peninsula revealed a large number with splenomegaly and resulted in the isolation of another less virulent isolate [15]. During the summer of 1971 in the Fairbanks area, 10 of 24 hares had enlarged spleens from which *F. tularensis *was isolated [16]. This isolate was compared to the vole isolate from 1963 [15] and shown to be significantly more virulent in challenge studies, further supporting the coexistence of type A and B strains in Alaska [16] (Table 1).

**Table 1:** Isolation of *Francisella tularensis *in Alaskan wildlife from 1938-1974

Year	Host	Location	# Positive	# Collected	Reference
1938	Rabbit Tick	Fairbanks	3 lots	3 lots	11
1953	Rabbit Tick	Minto, Livengood, Fairbanks	3 lots	14 lots	12
1960	Tick (from ptarmigan)	Livengood	1 lot	Unknown	24
1963	Red-backed vole	Alaska Peninsula	1	217	15
1971	Varying hare	Fairbanks	1	24	16

Though few isolates have been obtained, serological surveys for tularemia conducted between 1964 and 2000 have indicated the presence of *F. tularensis *among a wide variety of wildlife species and across a wide geographic area in Alaska. Seropositive animals (titer ≥ 1:20) in these surveys included various rodents and hares, birds and large predators (Table 2). Of those titers reported, the range was 1:20 - 1:320 [14,17-20]. These serology results are consistent with the wide number of species in which *F. tularensis *has been found [4], but revealed few clues as to the important reservoir(s) for *F. tularensis *in Alaska. Of note, however, were two studies by Zarnke et al. [19,20], which found that positive tularemia serology peaks in predators followed peaks in snowshoe hare populations, suggesting the possibility of a hare reservoir. In addition, *F. tularensis *DNA was found in 30% of > 2500 mosquitoes in Alaska, suggesting the possibility of an arthropod reservoir as well [21].

**Table 2:** Prevalence of *Francisella tularensis *antibodies (titer ≥ 1:20) in Alaskan wildlife from 1964 - 2000.

Year	Host	Location	#Positive	#Tested	Reference
1964	Dairy cattle	Tanana Valley	2	173	[14]
	Barrow ground squirrel	Tanana hills, Paxson	1	34	
	Red squirrel	Interior, Paxson	9	111	
	Red-backed vole	Interior, Paxson	2	120	
	Tundra vole	Interior, Paxson	11	229	
	Porcupine	Interior	1	2	
	Varying hare	Interior, Paxson	3	60	
	Cliff swallow	Interior	1	3	
	Bank swallow	Interior	1	38	
	Common redpoll	Interior, Paxson	1	15	
	Varied thrush	Interior	1	4	
	Northern water thrush	Tanana hills	1	3	
	American tree sparrow	Tanana hills	1	10	
	Willow ptarmigan	Tanana hills	1	2	
1967-68	Varying hare	Eagle	1	29	[18]
	Ground squirrel	Denali highway	2	72	
	Red-backed vole	Delta creek	1	376	
	Collared lemming	Nome	1	25	
	Wolf	Tok	1	15	
	Black bear	Circle hot springs	2	4	
	Marten	Eagle	9	26	
	Ermine	Katella	1	31	
	Lynx	Tok	1	4	
	Gray jay	Manley hot springs	2	19	
	Northern raven	Circle hot springs, Fairbanks	2	13	
	Northern shrike	Glenn highway	1	1	
1975-82	Wolf	Southcentral Alaska	16	67	[19]
1984-	Wolf	Southcentral Alaska	1	6	[20]
2000	Wolf	Central Interior	8	32	
	Wolf	Southern Interior	28	135	
	Wolf	Eastern Interior	2	30	
	Wolf	Western Interior	3	30	
	Wolf	Northern Interior	7	48	
	Wolf	Western arctic	5	75	
	Wolf	Eastern arctic	2	45	
1988-91	Grizzly bear	Kodiak island	3	77	[17]
	Grizzly bear	Alaska Peninsula	12	86	
	Grizzly bear	Interior Alaska	13	40	
	Black bear	Interior Alaska	13	40	
	Grizzly bear	Seward Peninsula	4	40	
	Grizzly bear	Noatak river drainage	12	87	
	Grizzly bear	Arctic northwest	34	96	
	Grizzly bear	Arctic northeast and central	15	54	

### History of human tularemia in Alaska

The first possible case of human tularemia in Alaska was reported in 1938 in a 62-year-old man from Wiseman, north of the Arctic Circle. The patient exhibited symptoms of ulceroglandular tularemia and was hospitalized for 2 months, though there was no laboratory confirmation of tularemia [13]. In 1946, a 31-year-old male from Northway (interior Alaska) with a history of skinning muskrats became the first laboratory-confirmed case by serology (titer 1:1280). His symptoms were headache, orbital pain, general aches and fever followed by development of swollen lymph nodes. The patient also reported that an ulcerated lesion had been present on his left middle finger for about one week. However, no isolate was cultured [22]. The first culture positive human infection occurred in 1974 in a 42-year-old laboratory worker with pneumonia whose pleural fluid yielded an isolate of *F. tularensis *[23].

Following the diagnosis of these initial cases of tularemia, surveillance projects were conducted throughout the state. The first of these occurred between 1954 and 1957 and involved 816 skin tests of inhabitants of Alaskan villages, of which 64 (8%) were positive, with 50 - 59 year olds having the highest incidence by age group [24]. The highest incidence was found in central Alaska, between Minto and Kaltag and as far north as Hughes, corresponding with the rich trapping areas in central Alaska. Following this initial surveillance, two additional surveys of Alaska Natives were completed. First, in the 1960s, serological surveys of 793 Aleut, Indian and Eskimo men showed an overall detection rate of 18% (139 of 793), with titers ranging from 1:20 to 1:640. A second survey involved skin tests on a subset of 115 (15%) of these Alaska Natives. Fifty-one (44%) of the 115 had positive skin tests, 43 (84%) of which also had detectable titers in the first survey, indicating a high correlation between skin test and titer results. Following these results, questionnaires were completed to determine if clinical disease resembling tularemia had been present. No difference in either total illness or tularemia-like illness was found between seropositive and seronegative groups, suggesting that the tularemia present in Alaska Natives may be of a less virulent type [25].

A final survey of Alaska Natives was completed in 1974. In this study, there were 4% (29 of 810) and 7% (28 of 402) positive titer rates (≥ 1:80, range 1:80-1:1280) in southwestern and east central Alaska, respectively. In addition, two seroconversions in children were documented (both > 4-fold increase in titer), with one child reporting a rash at around the time of the rise in titer and the other exhibiting no signs of disease. Similar to the previous surveys, no cases of tularemia-like illness were described in the villages studied, again suggesting that the tularemia present in these villagers was due to a less virulent type, that the route of infection favors asymptomatic disease or that Alaska Natives have developed resistance [23].

In 1993, two human cases related to housecats occurred in Fairbanks. One patient was a 44-year-old man who had been bitten on the thumb by his cat three and a half weeks prior to admission. Prior to the man's illness, his cat had been seen by a veterinarian and treated with antibiotics for an unknown febrile illness. The second patient was a 42-year-old veterinarian who presented with similar symptoms. The veterinarian had treated several cats with tularemia during the two-month period prior to his illness. Both human cases resolved with appropriate antibiotics [26].

Following the above housecat-associated cases, a serological survey of veterinarians was done in the Fairbanks area; two of 14 veterinarians (14%) had positive titers (≥ 1:80) for tularemia. Questionnaires sent to Fairbanks physicians and veterinarians indicated that 54% (15/28) and 92% (11/12), respectively, were aware that tularemia was prevalent in local wildlife. In addition, nine veterinarians had treated local domestic cats or dogs for suspected tularemia, indicating that household pets can pose a significant source for human infection [26].

### Epidemiology of reported human cases in Alaska 1946-2010

**Figure 1:**
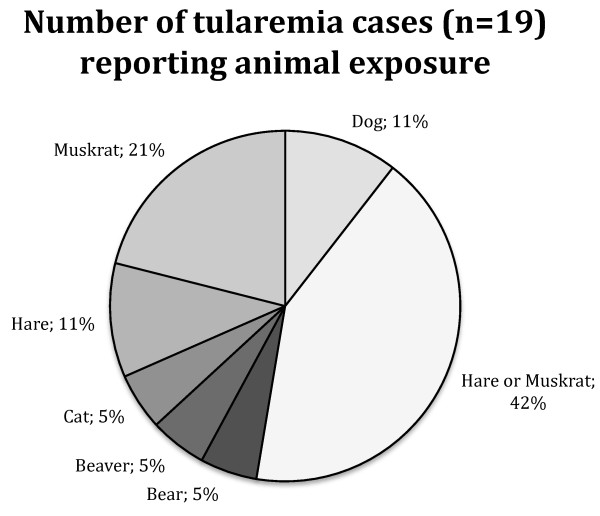
**Number of human tularemia cases in Alaska reporting animal exposure**.

Between 1946 and 2010, a total of 38 cases of tularemia were known to public health authorities in Alaska, with 9 cases in the Fairbanks-Steese area between 1946 and 1953 [27] and an additional 29 cases from throughout the entire state between 1972 and 2009. Of the 38 reported cases, 23 were laboratory confirmed, with detailed laboratory data available for 19 of those 23. Of these 19, 10 had four-fold changes in paired titers, 7 had positive cultures for *F tularensis*, 1 had a positive lymph node stain and 1 had a single high titer along with clinical and epidemiological evidence. Seventy-three percent (22 of 30) of the patients were male with a median age of 39 years (range of 15-59 years). Seventy-one percent (27 of 38) were white and 16% (6 of 38) were of unknown race. Most (69%, 20 of 29) had illness onsets between June and August. Geographically, 68% (26 of 38) were exposed in central eastern Alaska, 21% (8 of 38) in the greater Anchorage area, 5% (2 of 38) in northwestern Alaska, 3% (1 of 38) in Southeastern Alaska and 3% (1 of 38) were exposed out-of-state. Ulceroglandular tularemia was most common (70%, 19 of 27), followed by typhoidal (11%, 3 of 27) and pneumonic (7%, 2 of 27) tularemia. None of the cases were fatal. Of those case-patients with detailed exposure histories, 79% (19 of 24) had direct contact with animals and 84% (16 of 19) of those had contact with a known wildlife reservoir (Figure 1). The remaining 16% (3 of 19) had had contact with domestic animals (one cat bite and two dogs known to have killed hares).

### Molecular subtyping of recent *F. tularensis *isolates

We subtyped DNAs from eight recent (2003-2009) *F. tularensis *isolates (6 hare and 2 human) obtained by the public health laboratory of Alaska from interior Alaska and an additional four Alaskan DNAs (3 human and 1 rodent) available in Northern Arizona University's *F. tularensis *DNA collection to determine if the presumed coexistence of types A and B in Alaska could be confirmed. We first subtyped the isolate DNAs using a set of canSNPs described by Vogler et al. [28] to identify the major *F. tularensis *subclades found in Alaska. We then subtyped the isolate DNAs using the MLVA system described by Vogler et. al. [29] in order to identify additional variation among the isolates.

**Figure 2:**
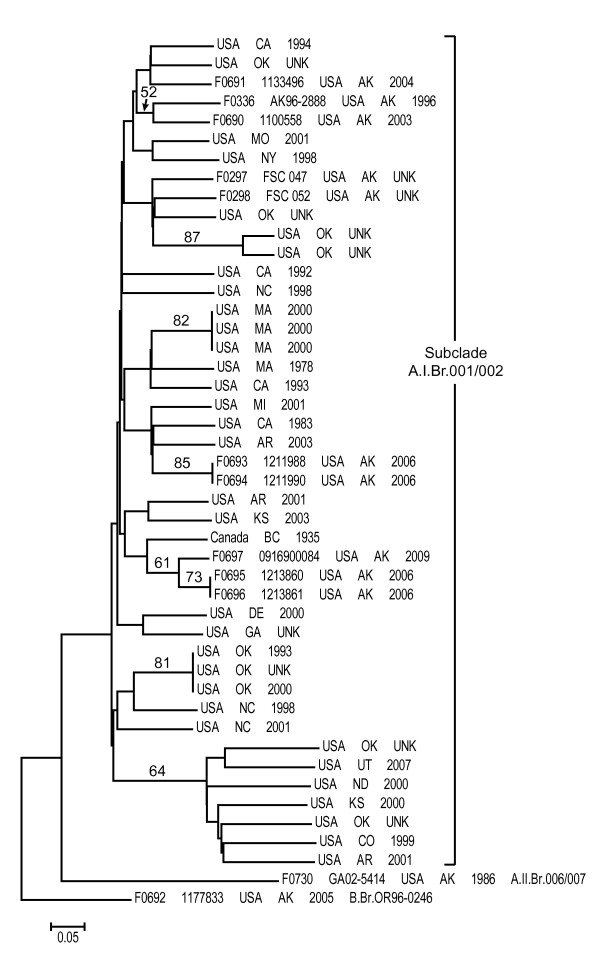
**Neighbor-joining dendrogram of Alaskan and 34 additional subclade A.I.Br.001/002 *F. tularensis *isolates based upon MLVA data**. The dendrogram was generated using neighbor-joining analysis of mean character differences using PAUP 4.0b10 (D. Swofford, Sinauer Associates, Inc., Sunderland, MA). Bootstrap values ≥50, also generated using PAUP 4.0b10, are indicated and were based upon 1,000 simulations.

The canSNP analysis identified 10 isolates as type A.I (6 hares, 1 rodent, 3 human), one as type A.II (human), and one as type B (human) (Figure 2). The canSNP assays further placed the type A.I isolates into subclade A.I.Br.001/002, the type A.II isolate into subclade A.II.Br.006/007 and the type B isolate into subclade B.Br.OR96-0246. This built upon a previous global analysis of *F. tularensis*, which had identified a single subclade A.I.Br.001/002 isolate (also included in this study) in Alaska [28]. This genetic analysis confirmed the previous virulence studies that had suggested the coexistence of types A and B in Alaska. Indeed, this analysis revealed an even greater level of genetic diversity than previously suspected, in that members of three major genetic groups were found to be present. The fact that most of these isolates were type A.I is likely related to the greater virulence of this genetic group [6] and thus the greater likelihood of severe disease and resultant opportunities for obtaining isolates through the public health system. However it is also possible that different strains are distributed differently throughout the environment, or that the reservoirs are distributed differently. It is probable that types B and A.II are present in much higher proportions in the wild than is indicated by this analysis. By relying on clinical isolates for genetic analysis we are limited to strains that are more likely to cause disease. Intensive sampling efforts would be needed to obtain more isolates from wildlife or people in the state.

The MLVA analysis revealed additional genetic diversity among the Alaskan isolates. Specifically, a neighbor-joining analysis based on MLVA data for the Alaskan isolates and an additional 34 A.I.Br.001/002 isolates revealed that the Alaskan subclade A.I.Br.001/002 isolates did not form a monophyletic group. Rather, they were scattered amongst subclade A.I.Br.001/002 isolates from diverse North American geographic locations (Figure 2), indicating a relatively high level of genetic diversity within this subclade in Alaska. This relatively high level of genetic diversity suggests either multiple introductions of *F. tularensis *to Alaska, a long history of *F. tularensis *in Alaska with ample time for diversification and possible transfers to other geographic locations, or a combination of the two. However, it is important to note that such high levels of genetic diversity within a single geographic location are not unique to Alaska, having been observed elsewhere in North America [28]. Additional whole genome sequencing, SNP discovery and SNP screening as well as increased sampling will likely be needed to determine the origins and spread of *F. tularensis *in North America as a whole and Alaska specifically.

Interestingly, though there was no obvious geographic separation among the different Alaskan subclade A.I.Br.001/002 MLVA genotypes as they were all collected from Interior Alaska, the single Alaskan type A.II isolate was geographically separated from the other Alaskan isolates. The type A.II isolate (human) was isolated from the Matanuska Susitna Valley whereas most of the other isolates were from interior Alaska, where most tularemia cases occur. These two regions are separated by the Alaska Range, which might serve as a geographic barrier separating type A.II *F. tularensis *from other *F. tularensis *genetic types in Alaska. However, this hypothesis would need to be confirmed by genotyping more isolates from both geographic regions.

## Conclusions

We have reviewed a history of *F. tularensis *in Alaska, beginning with its first isolation in a group of hare ticks in 1938 and progressing to its molecular characterization in 2011. Only limited studies have taken place within the state, there is still much to be learned about the ecology and epidemiology of tularemia, particularly in northern climates where it is endemic. We still do not know the reservoir in Alaska, though it is suspected to be hares or muskrats. We also do not know the prevalence of tularemia in most of the wildlife in the state. Overall the presented work suggests the need for renewed serological surveillance in both wildlife and humans to assess possible changes in Francisella prevalence in a rapidly changing Arctic. The current distribution of tularemia in Alaska is not well understood. While most cases are reported from Interior Alaska, while distribution of cases in wildlife or subclinical human cases is not known. In addition more molecular work is warranted to better understand the strains circulating in Alaska and assess potential for human infection associated with different host species. Transstadial transmission of tularemia should be addressed similar to work done in Sweden [30]. These steps will further increase our understanding of tularemia in Alaska and can guide public health surveillance and intervention.

## Competing interests

The authors declare that they have no competing interests.

## Authors' contributions

CH drafted the manuscript, AV DM and PK performed molecular analysis. KH conceived of the review and study. All authors have read and approved the final manuscript.
